# Evaluating masked self-supervised learning frameworks for 3D dental model segmentation tasks

**DOI:** 10.1038/s41598-025-01014-1

**Published:** 2025-05-14

**Authors:** Lucas Krenmayr, Reinhold von Schwerin, Daniel Schaudt, Pascal Riedel, Alexander Hafner, Marc Geserick

**Affiliations:** 1 Cooperative Doctoral Program for Data Science and Analytics, University of Ulm, 89081 Ulm, Germany; 2 Department of Computer Science, University of Applied Sciences, 89081 Ulm, Germany; 3 smyl tp GmbH, 89073 Ulm, Germany

**Keywords:** Computer science, Information technology, Image processing, Machine learning

## Abstract

The application of deep learning using dental models is crucial for automated computer-aided treatment planning. However, developing highly accurate models requires a substantial amount of accurately labeled data. Obtaining this data is challenging, especially in the medical domain. Masked self-supervised learning has shown great promise in overcoming the challenge of data scarcity. However, its effectiveness has not been well explored in the 3D domain, particularly on dental models. In this work, we investigate the applicability of the four recently published masked self-supervised learning frameworks-Point-BERT, Point-MAE, Point-GPT, and Point-M2AE-for improving downstream tasks such as tooth and brace segmentation. These frameworks were pre-trained on a proprietary dataset of over 4000 unlabeled 3D dental models and fine-tuned using the publicly available Teeth3DS dataset for tooth segmentation and a self-constructed braces segmentation dataset. Through a set of experiments we demonstrate that pre-training can enhance the performance of downstream tasks, especially when training data is scarce or imbalanced—a critical factor for clinical usability. Our results show that the benefits are most noticeable when training data is limited but diminish as more labeled data becomes available, providing insights into when and how this technique should be applied to maximize its effectiveness.

## Introduction

The utilization of three-dimensional (3D) dental models has become increasingly popular in dentistry and orthodontics for diagnosis, treatment planning of tooth misalignments, and the manufacture of dental restorations. These dental surface models are obtained by scanning physical impressions (i.e., plaster models) or nowadays, by advanced intraoral scanners (IOSs) that directly reconstruct the digital surface model of the dentition^1^. These dental models serve as key input for various computer-aided treatment planning applications. Modern software frequently employs a range of deep learning techniques to optimize the process of developing a treatment plan and automate tasks that would otherwise be labor intensive. For example, common applications include automatic tooth segmenting^2,3^, identifying important landmarks^4^, and completing faulty scans^5^.

**Fig. 1:**
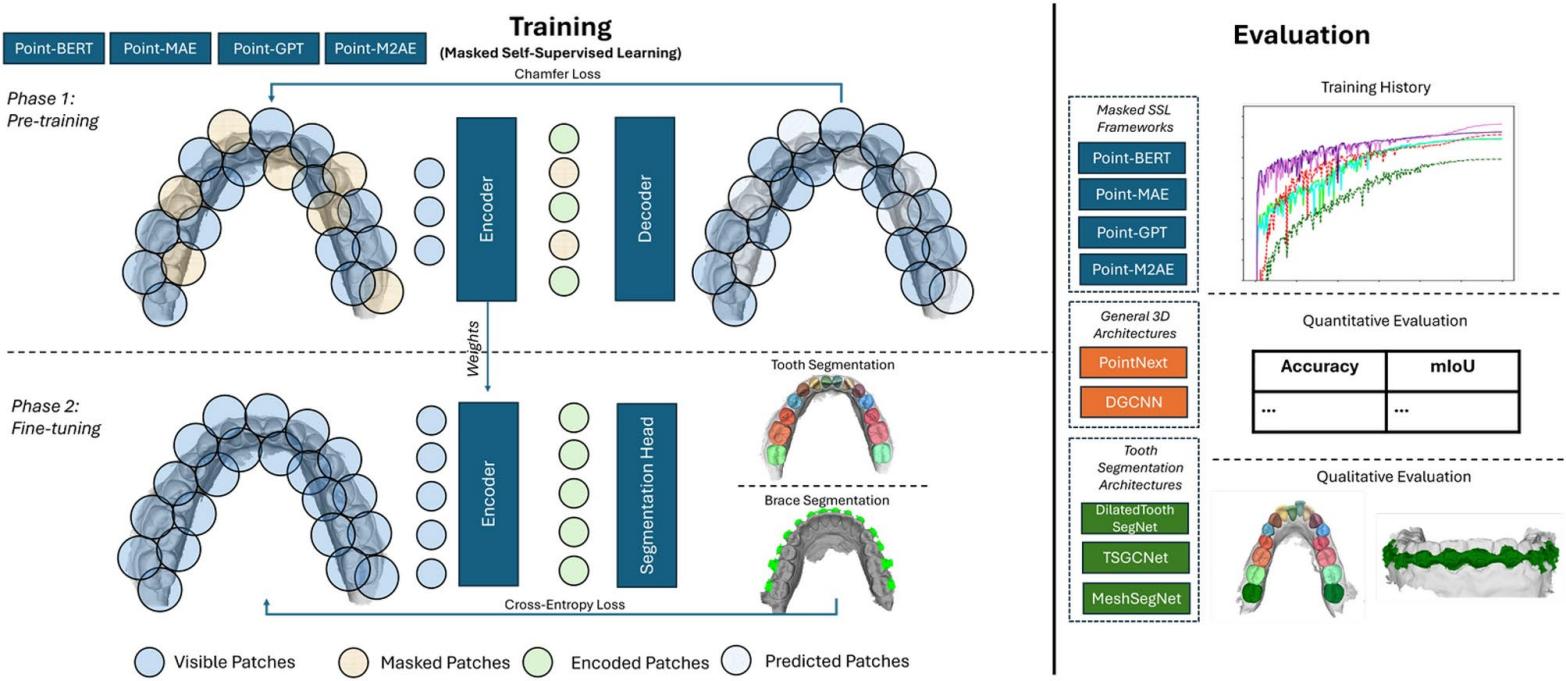
Graphical abstract: This work assesses four different frameworks for self-supervised learning on 3D dental models. All frameworks are based on a masked auto-encoder where the target is to reconstruct masked point-patches. In the downstream phase, the frameworks are evaluated on the tooth segmentation and brace segmentation tasks. Subsequently, they are evaluated by examining the training history and compared with traditional architectures for 3D data learning, as well as specialized architectures for tooth segmentation, through both quantitative and qualitative assessments.

Training these deep learning models in a supervised manner requires large amounts of labeled data. Obtaining this data in fields like medicine can be challenging, due to stringent data protection regulations and the complex, time-consuming nature of data labeling, which often requires medical expertise. An approach to address this issue is through the use of self-supervised learning (SSL)^6^. SSL focuses on learning latent features from unlabeled data rather than relying on human-provided annotations^7^. In the area of natural language processing (NLP), SSL is commonly applied, where large language models are trained on vast quantities of unlabeled textual data^8^. The objective here is to train a model to predict the next word within a sequence of input words, aiming to enrich the model’s understanding of natural language. Subsequently, this learned representation is fine-tuned for specific tasks. This concept has also been extended to the field of computer vision by training a masked auto-encoder based on a vision transformer (ViT), which attempts to reconstruct the original image given an image where certain areas (patches) have been masked^9,10^. This idea has been applied to the 3D domain as well. Here, the objective is to mask specific patches of a point cloud and train a model to reconstruct the original point cloud. The frameworks Point-BERT^11^, Point-MAE^12^, Point-M2AE^13^ and Point-GPT^14^ introduce different concepts that are all designed for masked self-supervised learning on point cloud data. Applying these methods to the field of dental medicine, which frequently faces the challenge of limited data and labor-intensive manual data annotation, could be highly beneficial. Thus, this work evaluates the effectiveness of these methods when they are trained on 3D dental models for specific tasks such as tooth segmentation (see Fig. 1). To achieve this, we leverage an unlabeled dataset consisting of over 4,000 3D dental models and fine-tune the networks using the publicly available tooth segmentation benchmark dataset Teeth3DS^15^ and a self-constructed dataset for braces segmentation.

Overall, our main contributions can be summarized as follows:

- Evaluated four masked self-supervised learning frameworks (Point-BERT, Point-MAE, Point-GPT, and Point-M2AE) on 3D dental models.
- Demonstrated the efficacy of SSL pre-training in improving performance on downstream tasks, such as tooth and brace segmentation, particularly in scenarios with limited labeled data.
- Showcased the practical use of SSL for tasks in orthodontics-related treatment planning by improving data efficiency and segmentation accuracy, but also highlighting the limitations of SSL by showing diminishing benefits as labeled training data increases.
- Provided insights into practical configurations (e.g., masking ratios and group sizes) for optimizing the use of these frameworks in the 3D dental domain.

## Related work

### Deep learning on 3D dental models

Deep learning is nowadays frequently applied in the dental field for computer-aided treatment planning, with a notable focus on 3D dental models. One common application involves the semantic segmentation of teeth and gums within these models. Early methods relied on geometric properties and harmonic field-based techniques^16–18^, requiring manual input and providing only a partially automated segmentation. Recent advancements in deep learning have addressed these limitations by enabling fully automated segmentation using CNNs to process dental model data directly through the conversion of the model into multiple 2D views^19^ or voxelization via octree partitioning^20^. However, these approaches may require additional preprocessing steps, potentially leading to information loss and quantization errors. Recent progress has explored the utilization of raw surface data from IOSs directly using point cloud or graph based neural network architectures^2,3^.

Another application involves the identification of anatomical landmarks on dental model surfaces, with neural networks trained to regress landmark heatmaps for landmark localization^4,21^. More advanced applications, such as automated tooth arrangement, aim to model the non-linear relationship between tooth features and transformation matrices to achieve automated tooth alignment^22^. Recently, the Transformer architecture has also been increasingly employed in this area^23^. However, due to its data-hungry nature, it is challenging to use it with limited amounts of labeled data. SSL offers a methodology to address this challenge.

### Masked-self-supervised learning on point clouds

Deep learning models often rely on vast amounts of labeled data for effective training. However, acquiring high-quality labeled data can be expensive and time consuming. SSL offers a compelling alternative by enabling models to learn meaningful representations from unlabeled data. At its core, SSL involves creating a pretext task that encourage the model to learn meaningful representations of the input data^24^. These pretext tasks typically include some form of data transformation or context prediction, where the model is tasked with predicting certain parts of the input data based solely on other parts. By formulating pretext tasks that are inherently related to the structure or semantics of the data, the model learns to extract high-level features that capture useful information about the input domain.

Self-supervised learning has shown significant success in various domains, including computer vision^9^, natural language processing^8^, and speech recognition^25^. By enabling models to learn from large amounts of unlabeled data, SSL holds the potential to overcome data scarcity issues and alleviate the need for extensive manual annotation. Moreover, the learned representations from self-supervised pre-training can be transferred to downstream tasks with limited labeled data, leading to improved performance and generalization. Recently, a range of architectures leveraging the self-supervised learning paradigm has been proposed to learn representations from point cloud data. However, these approaches are primarily evaluated on datasets such as ScanObjectNN^26^ and ModelNet40^27^, which consist of relatively simple everyday objects. In this work, we evaluate four transformer-based models for self-supervised learning on point clouds, with a specific focus on 3D dental models. The models follow a masked autoencoder approach where the pretext task is to reconstruct the original dental model from a partially masked version. All evaluated methodologies employ a three-step approach in the encoder stage: 1. partitioning of the point cloud into patches, 2. masking selected patches using a trainable token, 3. embedding the patches for further processing.

#### Point cloud partitioning

To create meaningful patches, these approaches employ two complementary algorithms for point cloud partitioning. Given an input point cloud with *p* points, denoted as $X \in \mathbb {R}^{p\times 3}$, a subset of *n* representative points are selected as patch centers using Farthest Point Sampling (FPS) (Eq. 1). FPS ensures an even distribution of centers across the point cloud, thereby capturing diverse regions of the object:

1 $$\begin{aligned} CT = FPS(X), \quad CT \in \mathbb {R}^{n\times 3} \end{aligned}$$

Next, local patches around each sampled center are defined. To achieve this, the k-nearest-neighbors (KNN) algorithm is applied to identify the *k* closest points from the original point cloud for each center. This results in a set of point patches, represented as:

2 $$\begin{aligned} P = KNN(X,CT), \quad P \in \mathbb {R}^{n\times k\times 3} \end{aligned}$$

To simplify, FPS identifies evenly distributed locations across the point cloud, while KNN gathers local neighborhoods around these locations to form patches. Within each patch, point coordinates are normalized relative to their center point to ensure consistent representation.

#### Masking and embedding

The generated patches serve as the ground truth representation, denoted as $P_{gt} \in \mathbb {R}^{n \times k\times 3}$. These patches are subsequently embedded into tokens using an encoder network, such as PointNet^28^, generating token representations $T_{e} \in \mathbb {R}^{n \times C}$, where *C* is the embedding dimension. Unlike standard Vision Transformers, which employ trainable linear projections, this approach ensures permutation invariance by leveraging a learned permutation-invariant feature extractor^12^. A transformer-based encoder–decoder architecture is then used to process the tokenized representations. During this stage, a fraction of the patches (determined by masking ratio *m*) is replaced with a learnable mask token, forming the partially masked token set $T_{d} \in \mathbb {R}^{n \times C}$. A prediction head, implemented as a fully connected network, reconstructs the original masked patches, yielding the predicted point cloud $P_{pre} \in \mathbb {R}^{n \times k\times 3}$. To guide the network in the pre-training stage, the Chamfer distance^29^ (Eq. 3) is used, which is a standard metric to measure the shape dissimilarity between point clouds, as well as a loss function for point cloud reconstruction tasks.

3 $$\begin{aligned} L = \frac{1}{\left| P_{pre} \right| } \sum _{a \in P_{pre}} \min _{b \in P_{gt}} \left| a-b \right| {2}^{2} + \frac{1}{\left| P{gt} \right| } \sum _{b \in P_{gt}} \min _{a \in P_{pre}} \left| a - b \right| _{2}^{2} \end{aligned}$$

#### Downstream applications

The learned representations can be fine-tuned for downstream classification and segmentation tasks. For classification, global pooling is applied to the patch embeddings, aggregating features into a compact representation, which is then passed through a MLP for classification. In segmentation, features from different transformer layers are concatenated, and local pooling is performed to obtain patch-wise features. These features are then upsampled using PointNet++’s feature propagation technique^30^, which interpolates local patch features back to the original point cloud resolution via inverse distance weighting and learned point-wise MLPs.

All selected frameworks adhere to the principles outlined. Nevertheless, the specific approaches also suggest various enhancements, which are briefly described in the following. For a more in-depth explanation, see the respective publications.

#### Point-BERT

Point-BERT^11^ incorporates the BERT training paradigm^8^ into the 3D domain. Inspired by BERT^8^, the authors follow a masked point-patch modeling task for the pre-training of a point cloud transformer. Initially, they acquire a point cloud tokenizer by training a discrete variational Auto Encoder (dVAE)^31^, which is used to convert point patches into discrete point tokens. Subsequently, they train a transformer backbone using the randomly masked point token representation to reconstruct the original point cloud. They also add an auxiliary contrastive learning task to help the transformer capture high-level semantic knowledge.

#### Point-MAE

Point-MAE^12^ builds upon the Point-BERT architecture but simplifies some core components by using a lightweight PointNet^28^ to encode each point-patches instead of having to train a separate dVAE tokenizer. This allows for a more efficient pre-training. In addition, they introduce a shared learnable token that is only used in the decoder part as also introduced by the original MAE architecture^9^.

#### Point-GPT

Point-GPT^14^ introduces the GPT concept to point clouds by arranging the point patches on a geometric ordering, namely the Morton-order curve^32^. Also, they introduce a dual masking strategy which additionally masks attending tokens for each token, which shall provide a more challenging pre-training task that demands comprehensive understanding.

#### Point-M2AE

Point-M2AE^13^ introduces a multi-scale MAE pre-training framework. Unlike previous frameworks, they introduce a pyramid architecture for the encoder and decoder. The framework uses a multi-scale masking strategy to progressively model spatial geometries and capture both fine-grained and high-level semantic features.

## Materials and methods

### Data and pre-processing

In this work, we use digitized 3D dental models of the maxilla and mandible, captured by IOSs, for both the pre-training and the downstream phase. These models are represented as triangular meshes, a type of polygon mesh composed of interconnected triangles^33^. Each face of these models forms a flat surface by connecting three vertices. In the following, we describe the specific details for the different phases and tasks.

#### Pre-training

**Fig. 2:**
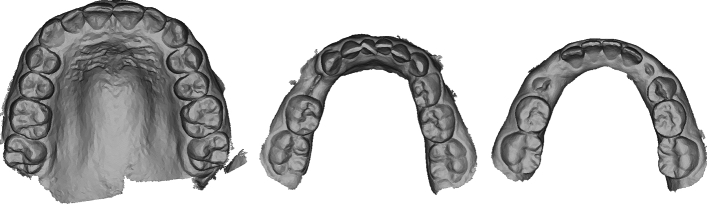
Examples from the dataset used for pre training. On the left a scan of the mandibular. In the center and on the right scans of the maxillary. All examples have a different anatomical structure in terms of the number of teeth and tooth alignment.

For pre-training, a proprietary dataset consisting of over 4,000 3D dental models was used. This dataset includes maxillary and mandibular dental models from various cases with different anatomical structures (see Fig. 2). The aim is to encompass a broad range of cases that reflect anatomical peculiarities.

#### Downstream

**Fig. 3:**
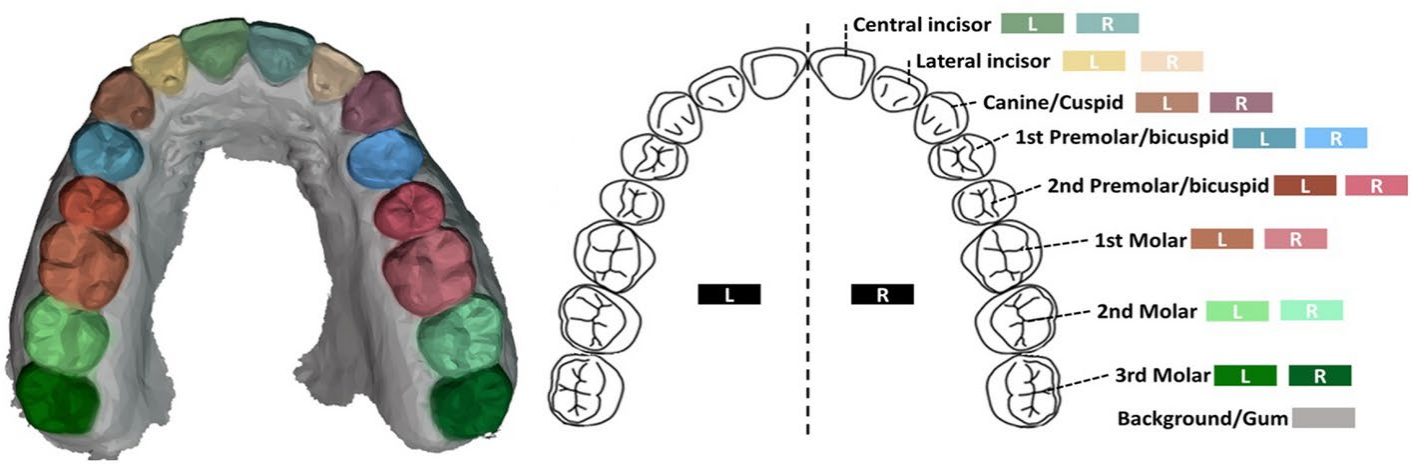
An example of a labeled dental model captured by an IOS containing all 16 teeth in the corresponding colors (left). An image of a dental arch including the tooth description and the corresponding color for the left and right quadrants (right). Reproduced from^2^ under the terms of the Creative Commons Attribution License (CC BY 4.0).

For the downstream task, two different datasets were used. The first dataset is Teeth3DS^15^, a benchmark dataset designed for teeth segmentation and labeling. Recently introduced at the MICCAI 2022 conference, Teeth3DS contains 1800 unique raw maxillary and mandibular dental surfaces obtained directly via IOS scans from 900 different patients. This dataset comprises 17 classes, including 16 tooth classes and one class for gums/background. An example of a labeled dental model along with its corresponding color encoding, including the scientific description per tooth, is presented in Fig. 3. According to the publisher, the dataset has been carefully validated by orthodontists and dental surgeons with more than 5 years of professional experience.

**Fig. 4:**
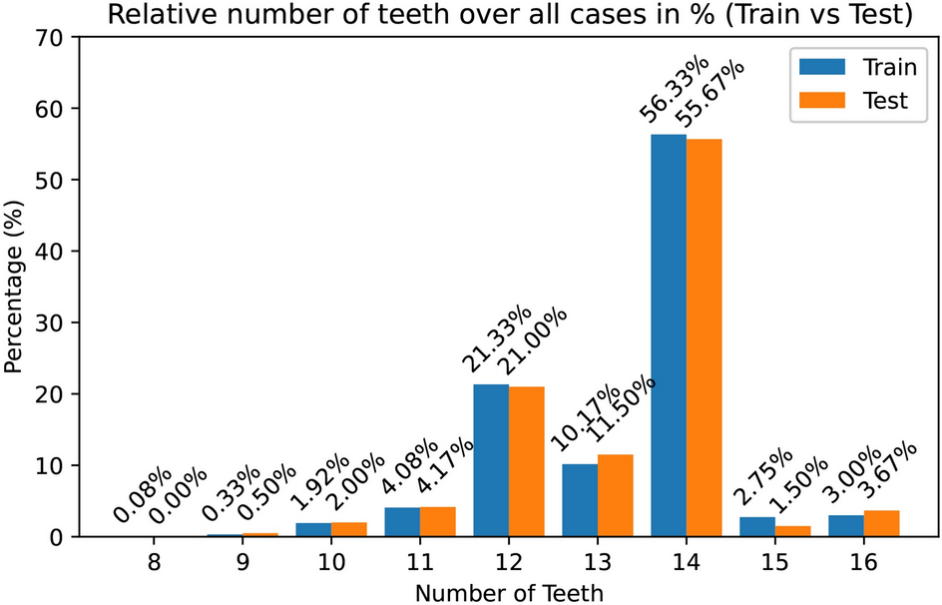
Relative number of teeth over all cases separated by the train and test set.

Figure 4 provides a statistical insight into the distribution of the number of teeth present. It presents an overview of the relative distribution of the number of teeth per case within the entire dataset. It is noticeable that the most common scenario is when the patient has 14 teeth. However, there are cases where the presence of the 3rd molars results in 15 or 16 teeth. In contrast, there are also several cases in which individual teeth are missing.

**Fig. 5:**
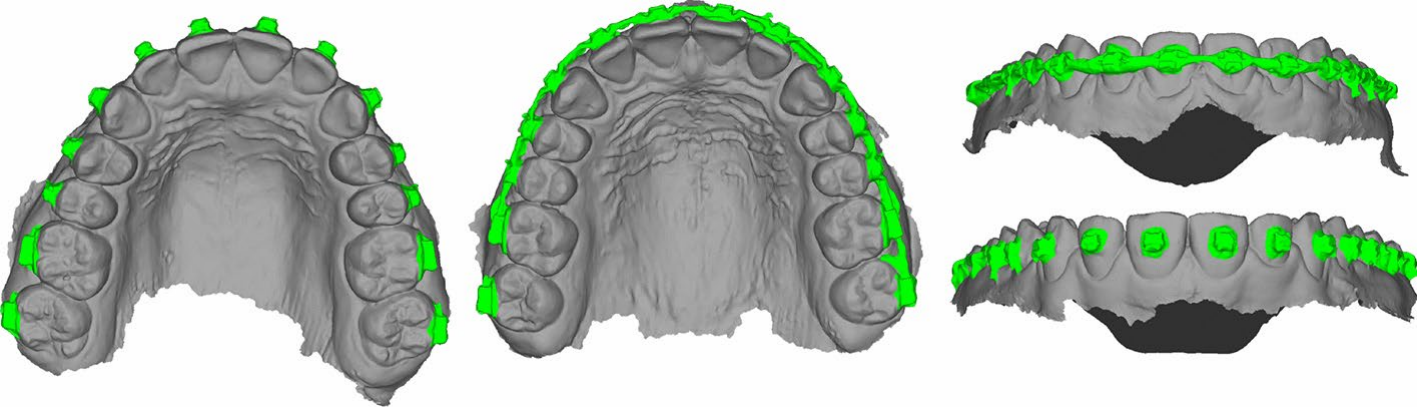
An example of a labeled 3D tooth model captured by an IOS containing a brace. The color indicates whether a particular region belongs to the patient’s anatomy or to the braces.

For our second dataset, we use a self-constructed small-scale dataset that contains 52 IOS scans of 26 patients with braces included in the digital scans. This dataset represents a binary segmentation problem in which the target is to detect areas containing braces. The ground truth is a binary segmentation mask, indicating whether a particular area belongs to the braces or to the rest of the model. An example is presented in Fig. 5, where two cases, from the top and the front, are visualized along with the segmentation mask.

Using this dataset, the objective is to train a network to predict a binary segmentation mask that indicates whether a particular region belongs to the patient’s anatomy or is the fragment of a brace. For training the network, the dataset is split into 40 training cases and 12 test cases.

#### Pre-processing and augmentation

Both the dataset for pre-training as well as the dataset for fine-tuning undergo the same pre-processing steps: The original dental surfaces have varying resolutions, typically containing several hundred thousand faces. To prepare them for training, a common approach involves down-sampling or down-scaling the meshes to a standardized size, as also done by others^2,3,34^. In this work, the meshes are simplified to a uniform size of $p=16,384$ faces using the quadric-based edge collapse simplification method^35^. The dental meshes with *p* faces are then converted to an input feature vector of $X \in \mathbb {R}^{p\times 3}$ where the features are the xyz coordinates of the center of each face. Following the approach of^2,3^. Furthermore, we apply z-score normalization^36^ to scale the coordinates to a standard scale.

To increase the variety of the datasets during training, we employ common data augmentation strategies to train neural networks on 3D data^37^. Specifically, the dental models are randomly rotated within the range of $-\frac{\pi }{6}$ to $\frac{\pi }{6}$ radians, scaled by a factor between 0.8 and 1.2 times their original size, and translated by a distance ranging from $-0.5$ to 0.5 units. This is intended to improve the model’s ability to generalize to new, unseen data by simulating a wider range of possible scenarios.

### Experiment setup

In the following we describe the experimental setup we used to evaluate these four frameworks—Point-BERT, Point-MAE, Point-GPT, and Point-M2AE—for masked self-supervised learning on dental models. The selection of these framekworks was based on several key considerations. First, these frameworks represent recent and widely recognized approaches to masked self-supervised learning on point cloud data. Second, they have demonstrated strong performance on standard point cloud benchmarks, suggesting their potential for learning geometric features - a crucial aspect when working with dental models where precise surface representation is essential. Third, their transformer-based architectures allow them to capture long-range dependencies in the data, which is particularly valuable for understanding the spatial relationships between different parts of dental structures, such as the arrangement of teeth and their relationship to surrounding anatomy. While these frameworks have shown promise in general point cloud tasks, their effectiveness in the specific context of dental models has not been previously evaluated, making this comparison particularly relevant for advancing computer-aided dental treatment planning.

Following the SSL methodology, the experiments are also divided into a pre-training and a downstream phase. All experiments were conducted on a Nvidia RTX 3090 GPU equipped with 24GB of memory using the deep learning framework PyTorch^38^.

#### Pre-training

In the pre-training phase we adhere to the procedure defined by the frameworks itself which is described in the previous section *Masked-Self-Supervised Learning on Point Clouds*. All frameworks follow the task of masked-self supervised learning by partitioning the input point cloud into patches by utilizing FPS to sample *n* patches followed by using KNN to group each patch with its *k* nearest neighboring points. A subset of these patches is then replaced with a learnable masking token, where the number of masked patches is determined by the masking ratio *m*. The target of the pre-training task is then to reconstruct the masked point patches given the unmasked point patches. The objective of the pre-training task is to reconstruct the masked point patches based on the unmasked ones. The network is optimized using the Chamfer distance (Eq. 3) as the loss function, guiding it to learn meaningful geometric representations.

The masking ratio *m* and the number of point patches *n*, along with their sizes *k*, are important hyper parameters guiding the network’s ability to learn semantically meaningful features from the pretext task. Therefore, different configurations were defined for the pre-training phase. The parameter number of groups (*m*) specifies into how many point patches the point cloud is divided, while the group size (*k*) defines the number of points each group contains. The configurations used in the experiments define four different configurations for the masking ratio, group size, and number of groups. They explore both low ($m=30$%) and high ($m=60$%) masking ratios, applied to different group sizes ($k=64$ and $k=256$) and corresponding numbers of groups ($n=512$ and $n=128$).

Following the approach of the frameworks authors, for pre-training, we used the AdamW optimizer^39^ in conjunction with a cosine learning rate decay strategy^40^. The initial learning rate was set to $1e-3$, with a weight decay of 0.05. Training was performed for a total of 300 epochs.

#### Downstream

The objective of this study is to assess whether pre-training enhances the performance of downstream tasks and how the resulting networks compare to commonly used architectures in 3D deep learning, or architectures particularly designed for supervised dental tooth segmentation. Hence, the in the 3D domain established DGCNN^41^ and PointNext^42^ architectures are included in the comparison. Additionally, we also evaluate the DilatedToothSegNet^2^, TSGCNet^3^ and MeshSegNet^34^ architectures, both of which are specifically designed for supervised dental tooth segmentation.

Unlike the presented transformer-based architectures, which subsample the point clouds by grouping neighboring points, these networks primarily utilize edge convolution to learn features at the face level. Notably, these models also incorporate normal vectors as additional input features. Concretely, both models expect an input feature matrix $X \in \mathbb {R}^{p\times 12}$, where the first 12 values encode the XYZ coordinates of the three vertices defining each face, along with the face center (3 $\times$ 3 + 1 $\times$ 3). The remaining 12 values represent the normal vectors of the three vertices and the face itself (3 $\times$ 3 + 1 $\times$ 3). This derivatives from the self-supervised frameworks evaluated in this work which rely solely on xyz coordinates as input features.

Additionally, we aim to evaluate how well the pre-trained networks perform with limited amounts of data compared to their non pre-trained counterparts. This is particularly relevant in the medical domain, where data is often limited and labeling data is complex and time-intensive. To simulate limited data, the networks were trained with only a subset of the Teeth3Ds dataset. In addition, the dataset introduced for brace segmentation contributes to this setup, being a small-scale dataset.

For fine-tuning on the tooth segmentation task, we employed the face-wise cross-entropy loss to guide the network during training, given the task involves multiple classes. Conversely, for braces segmentation, the face-wise binary cross-entropy loss was utilized, reflecting the binary setup of the segmentation task. Following the approach of the framework authors, fine-tuning was performed using the AdamW optimizer with a cosine learning rate decay strategy. The initial learning rate was set to $2e-4$, with a weight decay of 0.05. Training was performed for a total of 300 epochs. For all downstream experiments, we train the network three times with different seed values to mitigate the risk of random initialization affecting the results. Each training session is conducted under the same conditions, and the average performance across these runs is reported to ensure the robustness and reliability of the findings.

## Results

### Pre-training

Understanding the computational resources required in terms of memory and training time is essential to assess whether the potential benefits during the fine-tuning phase justify the additional effort. Table 1 presents the memory requirements, training time and the number of learnable parameters per framework with respect to the batch size used for pre-training. The goal was to maximize the use of available memory by maintaining the largest feasible batch size while ensuring comparability between the frameworks. However, the Point-BERT framework demanded substantially more memory, requiring a reduction of the batch size.

**Table 1:** Memory requirements, training time and number of learnable parameters per framework.

Framework	Configuration number of groups *n* group size *k*	Batch size	Memory required (GB)	Training time (h)	Nr of learnable parameters (M)
dVAE (Point-BERT)	$k=64$ $n=512$	22	24	8.5	28
	*k* = 256 *n* = 128	22	21	5.2	28
Point-BERT	*k* = 64 *n* = 512	18	24	11.2	25.5
	*k* = 256 *n* = 128	18	18	7	25.5
Point-MAE	*k* = 64 *n* = 512	26	23.5	5.5	29
	*k* = 256 *n* = 128	26	17.5	2.8	29
Point-GPT	*k* = 64 *n* = 512	26	22	8.2	29
	*k* = 256 *n* = 128	26	18.2	3.3	29
Point-M2AE	*k* = 64 *n* = 512	26	17	4	15.5
	*k* = 256 *n* = 128	26	16.5	3	15.5

Point-BERT requires the training of a dVAE as a prerequisite, which must be taken into account

It should be noted that the objective here was not to optimize memory consumption or training time. Instead, the implementations provided by the original authors were used as–is and adapted to the dataset. This table aims to provide an indication of the necessary requirements rather than precise numbers.

Based on the numbers presented, the following observations can be made:

- A larger number of groups leads to higher memory consumption. Smaller group sizes cannot compensate for this due to the quadratic complexity of the transformer’s attention mechanism^43^.
- Point-BERT, along with its prerequisite dVAE Tokenizer, has the most learnable parameters, resulting in the highest memory consumption. Consequently, the training time for Point-BERT is the longest due to substantial memory requirements and the necessity to train two networks.
- Point-MAE and Point-GPT have a similar number of learnable parameters and memory requirements. However, the training time for Point-MAE is slightly shorter.
- Point-M2AE has the lowest memory requirements and the smallest number of learnable parameters. Its training time is also comparable to that of Point-MAE.

Considering training time and memory requirements, the frameworks Point-MAE, Point-GPT and Point-M2AE provide an efficient architecture. Point-BERT is lagging behind due to the complexity introduced by the dVAE tokenizer.

### Downstream

In the downstream phase, we first analyzed the performance of the different frameworks using the Teeth3DS dataset for tooth segmentation. Subsequently, we investigated how pre-training affects the results in sparse data scenarios by limiting the available data and using the brace segmentation dataset. To evaluate performance, the mean Intersection over Union (mIoU) and accuracy was examined during the training phase.

#### Tooth segmentation

First, we conducted an analysis of the performance of the presented frameworks on the tooth segmentation task using different configurations. Additionally, the networks were trained from scratch (without pre-training) to evaluate the benefits of the pre-training phase. This analysis aims to provide insights into which configurations are most advantageous and how the different frameworks compare to each other in terms of accuracy.

**Fig. 6:**
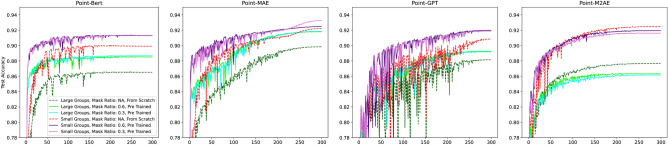
Average accuracy during training on the test set across three conducted training runs.

**Fig. 7:**
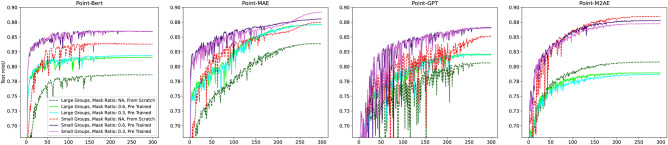
Average mIoU during training on the test set across three conducted training runs.

Figures 6 and 7 visualize the average face-wise accuracy and mIoU during training on the test dataset across three conducted training runs. The results are summarized in Table 2. The following key observations can be made:

- The frameworks Point-BERT, Point-MAE, and Point-GPT benefit from pre-training, achieving improved results compared to their counterparts trained from scratch.
- The Point-M2AE framework does not benefit from pre-training. In fact, the model trained from scratch achieves even better performance. This version achieves a notable accuracy of 92.47% and a mIoU of 88.52% resulting in the second best instance after the pre-trained Point-MAE.
- The size of the groups and the number of groups notable influence the results. Generally, smaller groups (64) and a larger number of groups (512) lead to improved performance across all frameworks. This can be explained by the fact that many smaller groups allow encoding of more detailed information, which is beneficial for highly accurate segmentation masks. However, this is accompanied by a higher computational effort.
- Point-MAE gains the most benefit from pre-training when using larger groups (256) but a smaller number of groups (128) with a lower masking ratio of 30%. This configuration results in an improvement of 2.04% in accuracy and an increase of 3.2 in mIoU.
- Among the evaluated frameworks, Point-MAE achieves the best performance, achieving an overall accuracy of 93.26% and a mean mIoU of 89.23%. This is obtained using smaller group sizes (64) and a larger number of groups (512) with a lower masking ratio of 30%. Compared to its non-trained counterpart, Point-MAE shows an improvement of 1.0% in accuracy and 1.73% in mIoU%.

**Table 2:** Average accuracy and mIoU for various configurations of masking ratio, group size, and number of groups, with and without pre-training across three conducted training runs.

Configuration				Accuracy				mIoU			
Masking ratio *m*	Group size *k*	Nr of groups *n*	Pre-trained	Point-BERT	Point-MAE	Point-GPT	Point-M2AE	Point-BERT	Point-MAE	Point-GPT	Point-M2AE
0.3	64	512	Yes	91.33	**93.26**	91.89	91.63	86.08	**89.23**	86.67	87.29
0.6	64	512	Yes	91.36	92.48	92.00	91.94	86.09	88.06	86.56	87.79
0.3	256	128	Yes	88.67	91.86	89.14	86.16	81.91	87.10	82.07	78.70
0.6	256	128	Yes	88.54	91.79	89.24	86.36	81.62	87.10	82.14	79.00
–	64	512	No	90.00	92.21	90.86	92.47	84.03	87.50	85.18	88.52
–	256	128	No	86.58	89.82	88.16	87.67	78.69	83.90	80.68	80.80

Best-performing results are in bold.

Given that the transformer architecture is relatively new in the field of 3D deep learning and there are already well-established architectures, the results were also compared with the PointNext^42^ and DGCNN^41^ architectures and the dedicated tooth segmentation architectures DilatedToothSegNet^2^, TSGCNet^3^ and MeshSegNet^34^. The results are summarized in Table 3, which presents the class-wise accuracy and mIoU for Point-MAE (both pre-trained and trained from scratch) and the competing methods. DGCNN is shown to perform the worst among the evaluated models. Also, when considering only the methods using xyz features, PointNext outperforms Point-MAE when trained from scratch. In contrast, the pre-trained Point-MAE surpasses the performance of the PointNext, demonstrating the advantages of pre-training. When including dedicated architectures using additional features, DilatedToothSegNet achieves the highest overall accuracy (OA) and mIoU. Nevertheless, the pre-trained Point-MAE exhibits only a marginal performance gap ($-0.62$% OA and $-0.22$% mIoU) and even outperforms DilatedToothSegNet in certain classes.

**Table 3:** Average class-wise accuracy and mIoU for Point-MAE (pre-trained and from scratch) and competing methods DGCNN and PointNext across three conducted training runs.

	Accuracy									
Model	Input features	OA	Gum	3rd M	2nd M	1st M	1st PM	C	LI	CI
DGCNN	Face xyz	84.07	81.51	33.65	57.92	76.11	74.31	73.76	75.56	58.44
PointNext	Face xyz	92.49	92.55	85.39	87.93	89.41	89.09	88.26	89.63	88.52
Point-MAE (from scratch)	Face xyz	92.21	91.44	79.82	87.37	89.37	89.54	88.84	88.88	87.45
Point-MAE (pre-trained)	Face xyz	**93.26**	**93.37**	**88.01**	**89.29**	**90.67**	**90.54**	**89.91**	**90.44**	**89.21**
*DilatedToothSegNet*	*Face and vertices xyz+normals*	*93.88*	*95.46*	*65.62*	*89.55*	*91.10*	*90.02*	*89.44*	*90.39*	*89.28*
*TSGCNet*	*Face and vertices xyz+normals*	*92.72*	*94.79*	*49.78*	*79.21*	*88.83*	*87.44*	*87.05*	*88.15*	*79.06*
*MeshSegNet*	*Face and vertices xyz+normals*	*93.48*	*94.37*	*73.16*	*88.92*	*90.77*	*90.48*	*90.30*	*90.14*	*89.10*

	mIoU									
Model	Data	OA	Gum	3rd M	2nd M	1st M	1st PM	C	LI	CI
DGCNN	Face xyz	77.41	71.39	27.39	49.62	63.79	63.58	63.19	63.56	50.19
PointNext	Face xyz	88.2	86.32	77.21	81.26	84.14	83.99	83.08	84.02	81.55
Point-MAE (from scratch)	Face xyz	87.50	85.00	73.06	79.84	83.35	83.90	83.35	83.26	79.82
Point-MAE (pre-trained)	Face xyz	**89.23**	**87.90**	**81.17**	**82.87**	**85.72**	**85.86**	**85.40**	**85.57**	**82.80**
*DilatedToothSegNet*	*Face and vertices xyz+normals*	*89.45*	*91.69*	*62.43*	*84.77*	*87.34*	*86.54*	*85.79*	*86.61*	*84.28*
*TSGCNet*	*Face and vertices xyz+normals*	*87.46*	*90.60*	*47.51*	*75.39*	*84.76*	*83.49*	*83.05*	*84.11*	*75.06*
*MeshSegNet*	*Face and vertices xyz+normals*	*89.29*	*90.66*	*68.67*	*84.44*	*87.30*	*87.14*	*86.64*	*86.51*	*84.30*

DilatedToothSegNet, TSGCNet and MeshSegNet are included as dedicated frameworks for dental tooth segmentation, which however use additional data. The metrics for the same tooth types from the left and right side are aggregated. Best-performing results using only xyz features are in **bold**. Overall best-performing results are underlined

Particularly notable are the class-wise results for the 3rd molar, commonly known as the wisdom tooth, which is underrepresented in the Teeth3DS dataset. The pre-trained version shows an improvement of 8.19% in class-wise accuracy and 8.11% in class-wise mIoU compared to the variant trained from scratch. Moreover, it outperforms DilatedToothSegNet, with gains of 22.39% and 18.74% considering the class-wise metrics for the 3^rd^ molar. This improvement is likely due to the more comprehensive representation of the dental anatomy acquired through pre-training, despite the limited instances of the 3^rd^ molar in the Teeth3DS dataset.

Overall, these findings highlight the effectiveness of pre-training, as the pre-trained Point-MAE competes with well-established architectures and approaches the performance of dedicated tooth segmentation networks, despite not relying on additional features or specialized architectural designs.

**Fig. 8:**
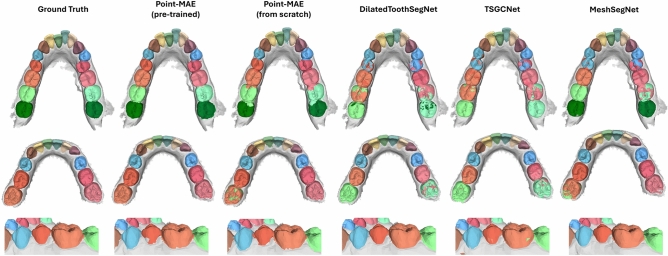
Visualization of three exemplary segmentation results by Point-MAE (both pre-trained and trained from scratch) and the dedicated architectures for tooth segmentation, DilatedToothSegNet, TSGCNet and MeshSegNet. The qualitative comparison indicates that especially the pre-trained Point-MAE effectively identifies underrepresented classes, such as the 3^rd^ (first example), and can handle cases of missing teeth (second example). However, it exhibits limitations in learning precise inter-class boundaries, where the dedicated architectures demonstrate better performance (third example).

Figure 8 additionally presents a visual comparison of segmentation results obtained using Point-MAE (both trained from scratch and pre-trained), DilatedToothSegNet, TSGCNet and MeshSegNet. This comparison highlights the strengths and limitations of the individual approaches, which are consistently observed across multiple examples in the dataset.

Point-MAE exhibits a strong global understanding of the dental structures, effectively segmenting underrepresented classes such as the 3^rd^ in the first example. This capability is likely due to the attention mechanism, which can capture long-range dependencies within the data. The pre-trained variant further enhances performance, confidently handling cases with missing teeth, as observed in the second example. In contrast, the version trained from scratch struggles to differentiate between the 2^nd^ and 1^st^ molar on the right side. The dedicated architectures, on the other hand, encounter difficulties in capturing global dependencies, leading to challenges in detecting the 3^rd^ molar in the first example or identifying the missing 2^nd^ molar in the second example. However, the third example illustrates a key limitation of Point-MAE, the imprecise inter-class boundaries. Even the pre-trained variant struggles to predict accurate boundaries compared to the dedicated architectures. A decisive reason for this is probably the use of normal vectors as additional input feature by DilatedToothSegNet, MeshSegNet and TSGCNet. These vectors are highly descriptive for boundary detection between teeth and gum as they precisely describe surface irregularities. Additionally, these architectures aggregate features at the face level across network layers rather than employing point cloud sub-sampling, as done in Point-MAE’s grouping process. This design enables them to learn highly descriptive face-level features, contributing to more precise segmentation boundaries. However, this results in increased computational complexity, making them challenging to scale and train.

#### Limited data

**Fig. 9:**
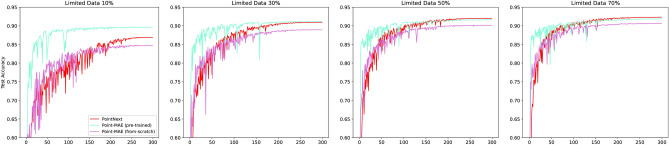
Average accuracy during training on the test set using limited amounts of training data across three conducted training runs.

**Fig. 10:**
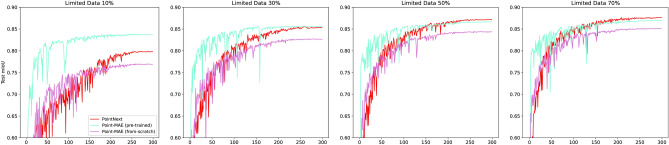
Average mIoU during training on the test set using limited amounts of training data across three conducted training runs.

Additionally, we examined the performance of the Point-MAE framework when the amount of training data is limited. Specifically, we trained both the pre-trained and non-pre-trained Point-MAE, as well as PointNext, using 10%, 30%, 50%, and 70% of the original dataset. Figures 9 and 10 illustrate the average accuracy and mIoU during training on the test dataset across three conducted training runs, while Table 4 outlines the results achieved based on the amount of training data. The key findings are as follows:

- The pre-trained Point-MAE converges after the least number of epochs compared to the other candidates. This offers the possibility of an early training stop.
- Under conditions of extremely limited data (10% and 30%), the pre-trained Point-MAE performs the best. Here, pre-training also provides the most notable performance boost compared to the version trained from scratch.
- When more data is available (50% and 70%), PointNext catches up and slightly outperforms the pre-trained Point-MAE.
- In all scenarios, pre-training enhances both accuracy and mIoU. However, the magnitude of these improvements decreases as the amount of available data increases. For instance, with only 10% of the data available, pre-training results in a 5% increase in accuracy and a 6.97% improvement in mIoU. However, with 70% of the data available, the improvements are only 1.09% in accuracy and 1.7% in mIoU.

**Table 4:** Average accuracy and mIoU for Point-MAE (pre-trained and from scratch) and PointNext across three conducted training runs performed with limited data.

Model	Accuracy	mIoU	Accuracy	mIoU	Accuracy	mIoU	Accuracy	mIoU
	10% Data 120 Instances		0% Data 360 Instances		50% Data 600 Instances		70% Data 840 Instances	
PointNext	86.89	79.87	90.84	85.54	**91.88**	**87.17**	**92.28**	**87.64**
Point-MAE (from scratch)	84.88	77.05	88.78	82.33	90.15	84.15	90.69	85.16
Point-MAE (pre-trained)	**89.88**	**84.02**	**91.17**	**85.78**	91.57	86.6	91.78	86.86

Best-performing results are in **bold**

In summary, pre-training leads to faster convergence and improved performance, particularly when training data is limited. However, with more data available, recent architectures such as PointNext achieve comparable results.

#### Braces segmentation

**Fig. 11:**
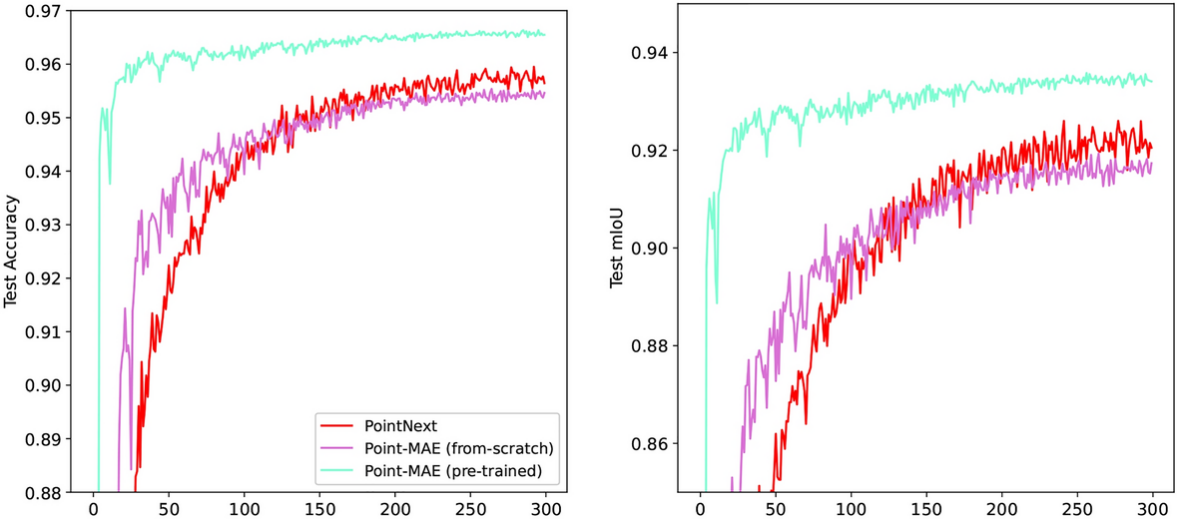
Average Accuracy and mIoU during training on the braces segmentation test set across three conducted training runs.

To further investigate the benefits of pre-training, we evaluated both the pre-trained and non-pre-trained versions of Point-MAE, as well as PointNext, on the brace segmentation dataset. The results are presented in Table 5 and Fig. 11. The findings indicate that the pre-trained Point-MAE variant converges significantly faster and achieves the best results. In this case, pre-training results in an average improvement of 1.08% in accuracy and 1.65% in mIoU. Additionally, the pre-trained variant demonstrates a more stable training progression compared to the non-pre-trained version.

**Table 5:** Average accuracy and mIoU for Point-MAE (pre-trained and from scratch) and PointNext across three conducted training runs using the braces segmentation dataset.

Model	Accuracy	mIoU
PointNext	95.94	92.6
Point-MAE (from scratch)	95.54	91.93
Point-MAE (pre-trained)	**96.62**	**93.58**

Best-performing results are in **bold**

**Fig. 12:**
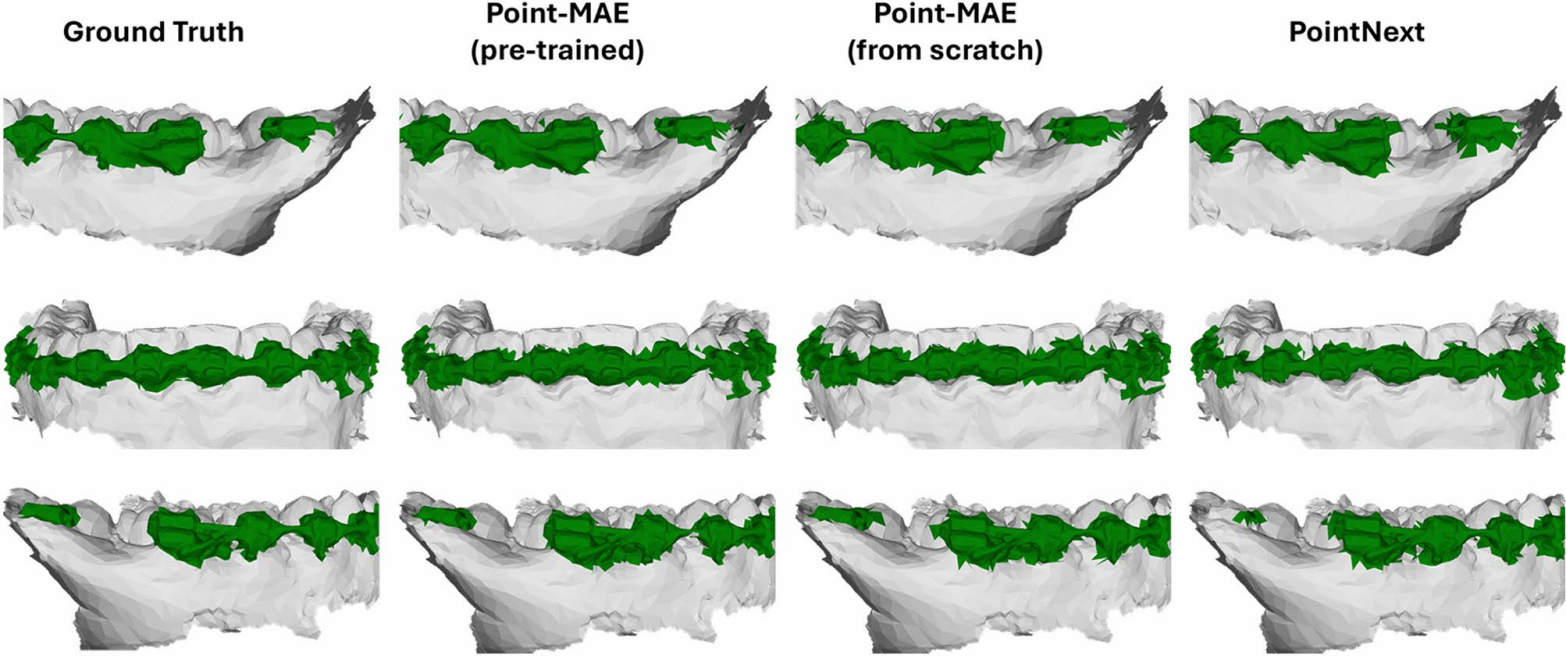
Visualization of three exemplary braces segmentation results by Point-MAE (both pre-trained and trained from scratch) and PointNext. Pre-trained Point-MAE exhibits slightly more precise segmentation boundaries, while the variant trained from scratch and PointNext produce noisier segmentation masks.

In addition to the quantitative results, Fig. 12 provides a qualitative comparison of three exemplary brace segmentation results. This figure presents the predicted segmentation mask by Point-MAE (pre-trained and trained from scratch) alongside PointNext. Overall, this visualization aligns with the quantitative results, by demonstrating that the different methods achieve comparable accuracy. However, the pre-trained Point-MAE exhibits slightly more precise segmentation boundaries, whereas variant trained from scratch and PointNext produce noisier segmentation masks with less defined boundaries.

#### Effects of pre-training

**Fig. 13:**
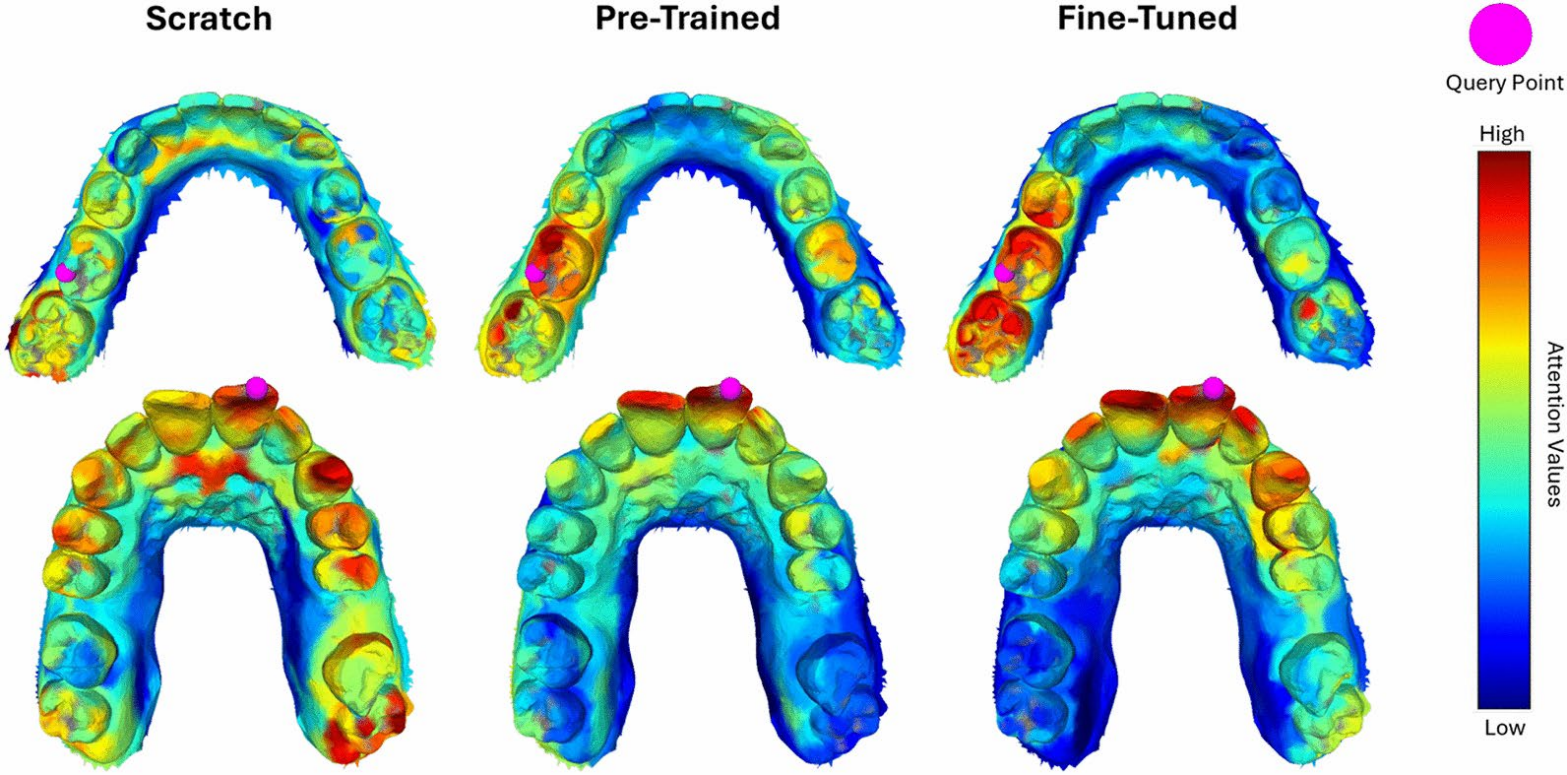
Visualization of the attention mechanisms learned by Point-MAE across three different setups. Left: Point-MAE trained from scratch on the Teeth3DS dataset. Middle: Point-MAE after the pre-training phase without fine-tuning. Right: Pre-trained Point-MAE fine-tuned on the Teeth3DS dataset. The pink sphere represents the query point, while the surface coloration encodes attention values, ranging from low (blue) to high (red).

Previous results illustrated both quantitatively and qualitatively that pre-training enhances performance in downstream tasks such as tooth or brace segmentation. Moreover, we analyzed the effect of pre-training on the model’s internal parameters. To assess this, we examined the learned attention values, which are often used as a strong indicator to explain how the network arrives at a prediction. Figure 13 illustrates the attention values for three different Point-MAE variants: (Left) Point-MAE trained from scratch on the Teeth3DS dataset, (Middle) Point-MAE immediately after pre-training without task-specific fine-tuning, and (Right) Point-MAE pre-trained using the masked reconstruction task followed by fine-tuning on the Teeth3DS dataset. The key observations are as follows:

- The model trained from scratch exhibits scattered and noisy attention patterns, suggesting a less focused learning of relationships between dental structures.
- The pre-trained variant shows more organized attention patterns, primarily focusing on surrounding teeth near the query point. This suggests a better understanding of structural dependencies.
- The fine-tuned variant builds upon the attention patterns learned during pre-training, retaining their structure while refining them to better align with the tooth segmentation task.

These observations suggest that training from scratch results in less meaningful attention patterns, likely due to the limited number of labeled samples available in the dataset. In contrast, pre-training on a larger and more diverse dataset enables the model to learn more informative attention patterns that capture relationships between dental structures. More specifically, the masked reconstruction task helps the network to learn data-dependent priors-such as leveraging the structure of unmasked regions of a tooth, as well as surrounding teeth, to reconstruct masked areas.

This prior knowledge is then transferred to the fine-tuning phase, where it is only slightly adapted to fit the specific requirements of the tooth segmentation task rather than being entirely relearned. Overall, these findings suggest that pre-training with the masked reconstruction task enables the learning of semantically meaningful features that are effectively transferred to the downstream task, thus enabling faster and more accurate task-specific learning.

## Discussion and conclusion

This paper evaluated four frameworks for masked self-supervised learning on 3D dental models: Point-BERT, Point-MAE, Point-GPT, and Point-M2AE. Our results demonstrate that Point-MAE offers a simple yet efficient architecture, achieving the best performance across various tasks and benefiting significantly from pre-training. This framework outperformed both its non-pre-trained counterpart and other established architectures like PointNext and DGCNN, particularly in scenarios with limited labeled data or underrepresented classes, such as the 3^rd^ molar. Additional comparisons with dedicated architectures for dental tooth segmentation like DilatedToothSegNet, TSGCNet and MeshSegNet, show that especially the pre-trained Point-MAE competes with these architectures despite not relying on additional features or specialized architectural designs. This highlights the promise of masked self-supervised learning in medical applications where labeled data is often scarce or imbalanced, as is the case with tooth segmentation.

Despite these benefits, the gains from pre-training diminish as the amount of labeled training data increases. While pre-trained models converge faster and require fewer training epochs, the marginal improvement over non-pre-trained variants and the substantial time required for pre-training may limit the practicality of this approach in some scenarios. Nonetheless, even modest improvements can be impactful in the medical domain. Also, a key advantage of pre-training is its ability to enhance model performance without the need for additional labeled data. Instead, unlabeled data, which is often collected during daily practice, can be leveraged for self-supervised learning. This makes pre-training particularly beneficial in domains where labeled annotations are scarce or expensive to obtain. Therefore, effective pre-training strategies, such as the one evaluated in this work, can significantly improve the practical usability and adoption of deep learning models in the dental domain.

Furthermore, the results demonstrate that the pre-trained Point-MAE exhibits an enhanced global understanding of dental structures. This suggests its potential as a powerful feature extractor, serving as a backbone for more sophisticated architectures in 3D dental model analysis. However, in our experiments, the conversion of 3D dental meshes to point cloud representations ignored surface information such as normal vectors, a feature frequently used by dedicated architectures for learning on dental models^2,3,34^. Our visual evaluation has shown that models utilizing surface normals can achieve more fine-grained segmentation masks. To address this limitation, in future work, we will incorporate normal vectors and evaluate their impact. Additionally, we aim to integrate the benefits of pre-training into a more advanced network architecture specifically tailored for dental model analysis. Beyond segmentation, we will extend our evaluation to other tasks, such as landmark prediction, thereby broadening the potential applications of our approach in dental care and treatment planning.

In this work, we primarily focused on evaluating self-supervised pre-training frameworks based on masked autoencoders, as these methodologies are also used in recent state-of-the-art vision foundation models. However, alternative self-supervised pre-training approaches exist in the 3D domain, such as PointContrast^44^, which utilizes contrastive learning. This idea has also been applied to dental data, as demonstrated by STSNet^45^, which employs contrastive learning on 3D dental models. Additionally, MGFLNet^46^ improves segmentation accuracy by incorporating self-supervised signals derived from a modified grid spectral clustering algorithm. Future work could extend this evaluation by exploring self-supervised learning methods beyond masked autoencoding, including mentioned approaches.

## Data Availability

The data used for pre-training and for the braces segmentation task are proprietary datasets and not openly available due to relevant data protection laws. A sample of the data will be made available upon reasonable academic request from the corresponding author. The data used for the tooth segmentation task is the publicly available dataset called Teeth3DS which is accessible here: https://github.com/abenhamadou/3DTeethSeg22_challenge.
